# Efficacy of endoscopic management for anastomotic leakage after gastrectomy in patients with gastric cancer

**DOI:** 10.1007/s00464-021-08582-z

**Published:** 2021-07-12

**Authors:** Young-Il Kim, Jong Yeul Lee, Harbi Khalayleh, Chan Gyoo Kim, Hong Man Yoon, Soo Jin Kim, Hannah Yang, Keun Won Ryu, Il Ju Choi, Young-Woo Kim

**Affiliations:** 1 Center for Gastric Cancer, National Cancer Center, 323 Ilsan-ro, Ilsandong-gu, Goyang, 10408 Republic of Korea; 2 The Department of Surgery, Faculty of Medicine, Kaplan Medical Center, Hebrew University of Jerusalem, Jerusalem, Israel; 3 Division of Biology and Biological Engineering, California Institute of Technology Pasadena, Pasadena, CA 91125 USA; 4 Department of Cancer Control and Population Health, Graduate School of Cancer Science and Policy, 323 Ilsan-ro, Ilsandong-gu, Goyang, 10408 Republic of Korea

**Keywords:** Anastomotic leakage, Endoscopic therapy, Gastrectomy

## Abstract

**Background:**

Anastomotic leakage (AL) after gastrectomy in gastric cancer patients is associated with high mortality rates. Various endoscopic procedures are available to manage this postoperative complication. The aim of study was to evaluate the outcome of two endoscopic modalities, clippings and stents, for the treatment of AL.

**Patients and methods:**

There were 4916 gastric cancer patients who underwent gastrectomy between December 2007 and January 2016 at the National Cancer Center, Korea. A total of 115 patients (2.3%) developed AL. Of these, 85 patients (1.7%) received endoscopic therapy for AL and were included in this retrospective study. The endpoints were the complete leakage closure rates and risk factors associated with failure of endoscopic therapy.

**Results:**

Of the 85 patients, 62 received endoscopic clippings (with or without detachable snares), and 23 received a stent insertion. Overall, the complete leakage closure rate was 80%, and no significant difference was found between the clipping and stent groups (79.0% vs. 82.6%, respectively; *P* = 0.89). The complete leakage closure rate was significantly lower in the duodenal and jejunal stump sites (60%) than esophageal sites (86.1%) and gastric sites (94.1%; *P* = 0.026). The multivariate analysis showed that stump leakage sites (adjusted odds ratio [aOR], 4.51; *P* = 0.031) and the presence of intra-abdominal abscess (aOR, 4.92; *P* = -0.025) were associated with unsuccessful leakage closures.

**Conclusions:**

Endoscopic therapy using clippings or stents is an effective method for the postoperative management of AL in gastric cancer patients. This therapy can be considered a primary treatment option due to its demonstrated efficacy, safety, and minimally invasive nature.

The incidence of gastric cancer remains significant in East Asian countries [1], but the mortality is decreasing due to a combination of nationwide gastric cancer screening programs and advancements in radical surgery, technology, and procedural techniques [2, 3]. However, anastomotic leakage (AL) is an alarming and life-threatening postoperative complication that is significantly associated with postoperative mortality in gastric cancer patients [4]. Approximately, 1.7 to 7.5% of all complications [5–9] and 20 to 75% of post-gastrectomy mortality (depending on the type of gastrectomy performed) [10–13] is attributed to ALs. Other than mortality, the consequences of AL include reduced digestive function and quality of life [14], in addition to increased hospital stay and financial burden [4, 15].

There are three therapeutic modalities to treat this condition: surgical reoperation of septic patients with early and acute AL, conservative management of late and asymptomatic AL patients, and endoscopic management for patients with severity between these two ends of the spectrum [16]. Reoperations were previously the method of choice for AL treatment but are associated with high rates of morbidity and mortality [7, 8]. Minimally invasive endoscopic intervention has continually gained evidence-based support [17–21], which has resulted in a paradigm shift toward endoscopic management of ALs in the past 20 years.

Out of the many emerging endoscopic strategies for the management of AL, two accepted and widespread applications are the use of clippings and stents. Favorable outcomes have been reported in serial case studies of patients who received different variations of these two techniques, including endoscopic clippings with or without detachable snares [18, 19], over-the-scope clip systems [20], and self-expanding stents [17, 18, 21]. However, these studies are limited by small case sizes (fewer than 30) or heterogeneous populations due to differing diagnoses necessitating gastrectomy or esophagectomy. As such, this retrospective study was conducted to evaluate the outcome and risk factors of endoscopic therapy for AL in a large cohort, which consisted of consecutive patients who underwent gastrectomy for gastric cancer.

## Patients and methods

### Patients

**Fig. 1:**
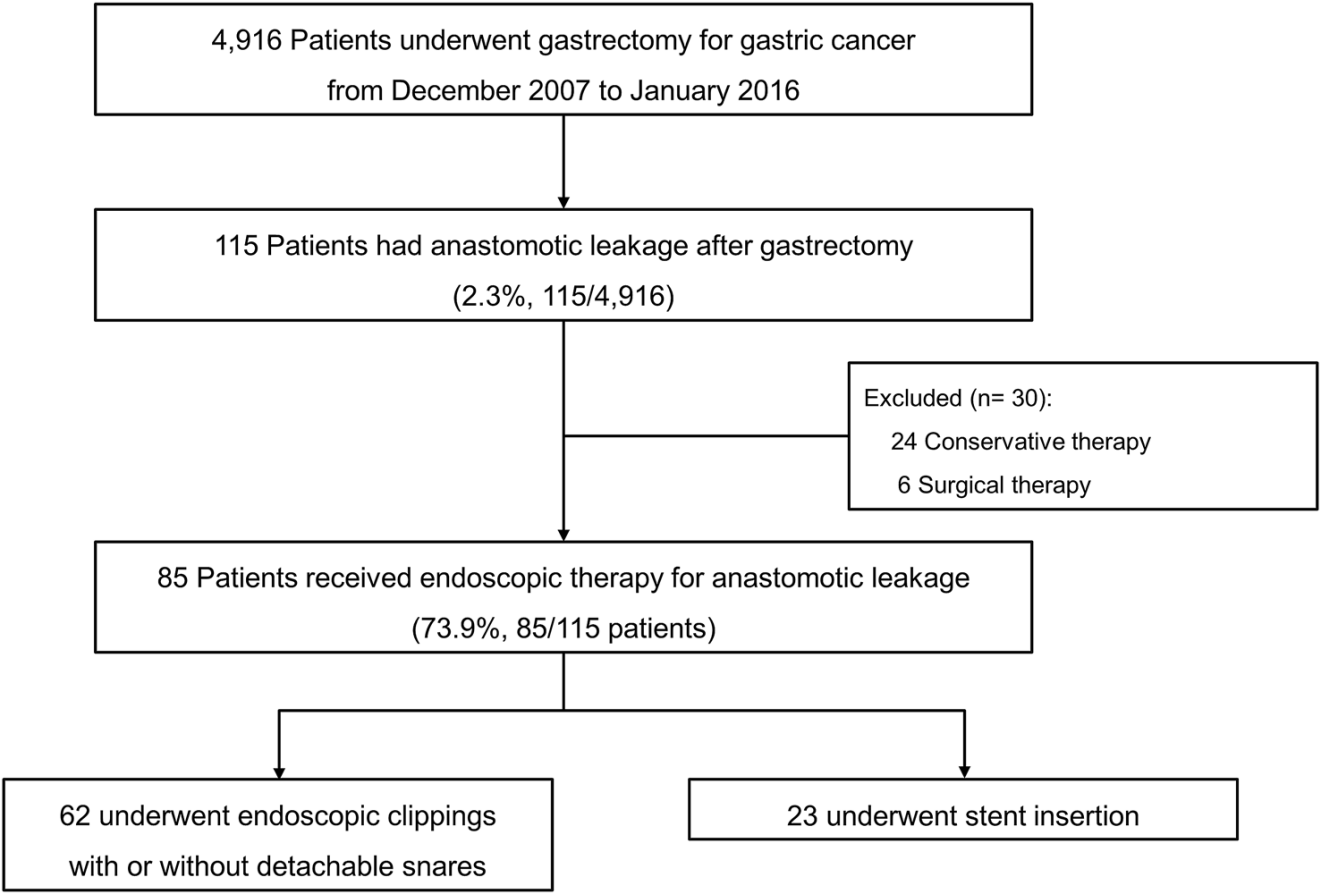
Study flows

Between December 2007 and January 2016, 4,916 patients who were diagnosed with gastric cancer underwent gastrectomy at the National Cancer Center, Korea. We reviewed the medical records of these patients, and those who developed AL after gastrectomy were considered for inclusion in this study. Of these patients, those with ALs that were managed conservatively or surgically were excluded so that only patients who underwent endoscopic treatment were included in the final analysis (Fig. 1).

Baseline demographic and clinical characteristics were obtained from a prospectively collected database, including age, sex, body mass index, comorbidities, American Society of Anesthesiologists (ASA) score [22], tumor characteristics, and method of gastrectomy. This study was approved by the Institutional Review Board (IRB) of the National Cancer Center, Korea (IRB approval Number, NCC2016-0229). The requirement for informed consent was waived for all subjects by the IRB because of the low-risk nature of the study.

### Surgical procedures for gastrectomy

All patients underwent total, subtotal, proximal, or pylorus-preserving gastrectomy with lymph node dissection performed by experienced surgeons. The following reconstruction methods were used: Roux-en-Y esophagojejunostomy for total gastrectomy; Billroth I, II, or Roux-en-Y anastomosis for subtotal gastrectomy; esophagogastrostomy for proximal gastrectomy; and gastrogastrostomy for pylorus-preserving gastrectomy. The extent of lymph node dissection was D1 + or D2 according to the Japanese gastric cancer treatment guidelines [23]. All gastrectomies were performed by laparoscopic or open surgery.

### Diagnosis of AL after gastrectomy

After gastrectomy, patients who had clinical presentations suggesting peritonitis and abnormally drained gastrointestinal contents were suspected of having AL. AL was confirmed in these patients if the leakage was confirmed by abdominal computed tomography (CT), fluoroscopy with radiocontrast, or endoscopy.

### Endoscopic management of AL

**Fig. 2:**
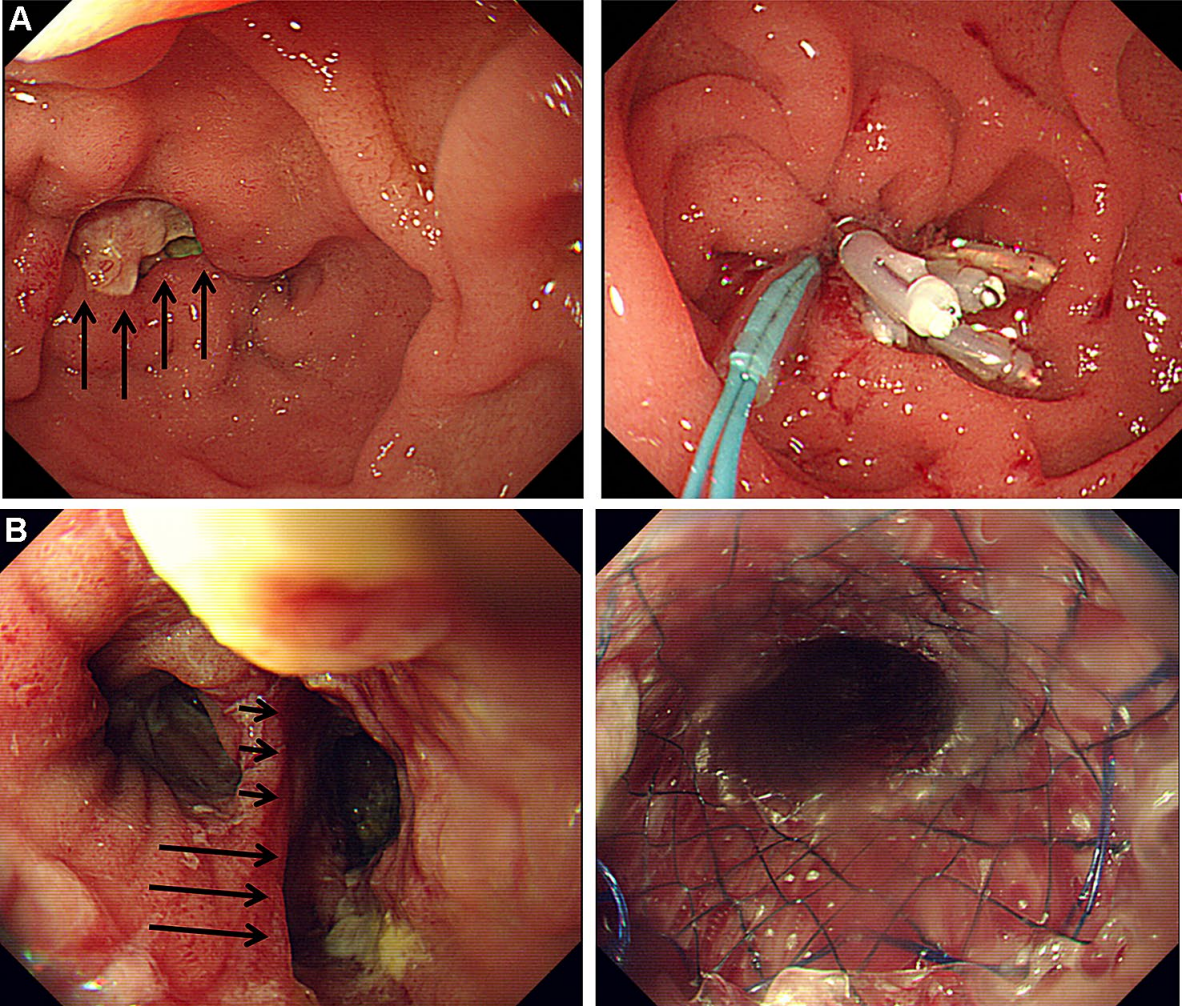
Endoscopic management of anastomotic leakage. **A** Endoscopic clippings with detachable snares were used to achieve primary closure for duodenal stump leakage after subtotal gastrectomy with Billroth II anastomosis (arrows). **B** Endoscopic stent insertion using Shim’s technique was performed to treat anastomotic leakage at the esophagojejunostomy site after total gastrectomy (arrows)

Endoscopic clippings or stents were used for the management of AL. Experienced endoscopists performed all procedures with a standard upper endoscope (GIF-2T240 or GIF-H260; Olympus, Tokyo, Japan). Endoscopic clippings of small defects were performed using direct closure with multiple hemoclips (EZ Clip_TM_, HX-610-090L or HX-610-135L; Olympus, Tokyo, Japan). In cases of major defects that could not be closed directly by hemoclips alone, detachable snares were used to approximate the size of the AL (Endo-Loop_TM_, MAJ-254 or MAJ-340; Olympus, Tokyo, Japan) after applying hemoclips around the margin of the AL (Fig. 2A).

For patients managed using stents, a modified fully covered self-expandable metal stent (SEMS) (CHOOSTENT_TM_; M.I.Tech, Suwon, Korea) was applied using Shim’s technique as described in a previous study [24]. After placement of the guidewire, the modified fully covered SEMS was implanted across the leakage site under fluoroscopic and endoscopic guidance. Finally, the SEMS was fixed in the appropriate position using a technique similar to that of pulling out a nasobiliary drainage catheter (Fig. 2B).

### Follow-up after endoscopic managements and study outcomes

Follow-up endoscopic examinations were performed every 3 days for patients who underwent endoscopic clippings and every 7 days for those who received a stent. In cases of persistent AL, additional endoscopic therapies were performed at the discretion of the endoscopist, including additional clippings and stent repositioning. After oral administration of radiocontrast, fluoroscopy or CT scans confirmed the complete closure of the AL. The primary outcome of this study was the efficacy of endoscopic management, which was defined as the rate of complete closure. Secondary outcomes were the differences in clinical factors associated with successful AL closure between the two methods of endoscopic management.

### Statistical analysis

The two endoscopic management groups were compared using the Chi-squared test or Fisher’s exact test for categorical variables and the Mann–Whitney *U* test for non-categorical variables. Factors associated with complete leakage closure were investigate using univariate logistic regression analyses. Covariates that were statistically significant in the univariate analyses were included in a multivariate logistic regression analysis. A *P* value of less than 0.05 was considered to be statistically significant. Data were analyzed using Stata 16.0 (StataCorp, College Station, TX, USA).

## Results

### Baseline characteristics

A total of 115 gastric cancer patients (2.3%, 115/4,916 patients) developed AL after gastrectomy between December 2007 and January 2016 (Fig. 1). Of these, 30 patients who received a conservative therapy or surgical therapy were excluded. Conservative therapy was performed in 24 clinically stable patients with AL only evident on abdominal CT scan. Six patients received surgical therapy because they had another severe condition including severe septic condition (*n* = 3), uncontrolled intra-abdominal bleeding (*n* = 2), and diffuse ischemic change of gastric wall (*n* = 1). Finally, the remaining 85 patients who received endoscopic therapy for AL (62 received endoscopic clippings with or without detachable snares [clipping group] and 23 received stents [stent group]) were included. The baseline clinical characteristics of the patients are presented in Table 1. Median age of the included patients was 63 years, and the proportion of male patients was 77.7%.

**Table 1:** Baseline clinical characteristics

Characteristics	Total *N* = 85	Endoscopic therapy		*P* value
		Clips ± snare *N* = 62	Stent *N* = 23	
Age (years), median (IQR)	63 (52–71)	61 (49–70)	67 (59–73)	0.038
Sex, *n* (%):				0.504
Male	66 (77.7)	47 (75.8)	19 (82.6)	
Female	19 (22.4)	15 (24.2)	4 (17.4)	
BMI (kg/m^2^), mean ± SD	24.3 ± 2.7	24.2 ± 2.6	24.7 ± 3.1	0.232
Comorbidity^a^, *n* (%)	45 (52.9)	30 (48.4)	15 (65.2)	0.167
ASA score, *n* (%)				0.628
1	22 (25.9)	15 (24.2)	7 (30.4)	
2	59 (69.4)	43 (69.4)	16 (69.6)	
3	4 (4.7)	4 (6.5)	0 (0)	
Cancer type, *n* (%)				0.014
EGC	48 (56.5)	40 (64.5)	8 (34.8)	
AGC	37 (43.5)	22 (35.5)	15 (65.2)	
Tumor location, *n* (%)				0.242
Upper	32 (37.7)	20 (32.3)	12 (52.2)	
Middle	25 (29.4)	19 (30.7)	6 (26.1)	
Lower	28 (32.9)	23 (37.1)	5 (21.7)	
Tumor stage^b^, *n* (%)				0.075
I	52 (61.2)	42 (67.7)	10 (43.5)	
II	14 (16.5)	8 (12.9)	6 (26.1)	
III	18 (21.2)	12 (19.4)	6 (26.1)	
IV	1 (1.2)	0 (0)	1 (4.4)	
Operation time (minutes), mean ± SD	222.5 ± 80.3	213.4 ± 73.9	247.1 ± 92.8	0.042
Type of gastrectomy, *n* (%)				0.038
Total	41 (48.2)	24 (38.7)	17 (73.9)	
Subtotal	34 (40.0)	29 (46.8)	5 (21.7)	
Proximal	3 (3.5)	3 (4.8)	0 (0)	
PPG	7 (8.2)	6 (9.7)	1 (4.4)	
Reconstruction method, *n* (%)				0.004
Billroth I	13 (15.3)	10 (16.1)	3 (13.0)	
Billroth II	20 (23.5)	19 (30.7)	1 (4.4)	
Roux-en-Y	45 (52.9)	26 (41.9)	19 (82.6)	
Others^c^	7 (8.2)	7 (11.3)	0 (0)	
Mode of surgery, *n* (%)				0.011
Laparoscopy	45 (52.9)	38 (61.3)	7 (30.4)	
Open	40 (47.1)	24 (38.7)	16 (69.6)	

*ASA* American society of anesthesiologists, *BMI* body mass index, *IQR* interquartile range, *PPG* pylorus-preserving gastrectomy, *SD* standard deviation

^a^Comorbidity included hypertension, diabetes mellitus, cardiac arrhythmia, ischemic heart disease, and liver cirrhosis

^b^The 7^th^ edition of the International Union Against Cancer/American Joint Committee on Cancer TNM classification system was used for gastric cancer staging

^c^Other anastomosis methods included esophagogastrostomy and gastrogastrostomy

Compared with the clipping group, the stent group was older (*P* = 0.038), had a higher proportion of advanced gastric cancer (*P* = 0.014), had longer surgery time (*P* = 0.042), and included more patients who underwent total gastrectomy (*P* = 0.038), open gastrectomy (*P* = 0.011), and Roux-en-Y anastomosis (*P* = 0.004). There were no significant differences in other baseline clinical characteristics, including sex, body mass index, comorbidities, ASA scores, tumor location, and stage.

### Characteristics of AL according to endoscopic management (Table 2)

**Table 2:** Comparisons of anastomotic leakage characteristics according to modalities of endoscopic therapy

Characteristics	Endoscopic therapy		*P* value
	Clips ± snare *N* = 62	Stent *N* = 23	
Diagnostic method of leakage, *n* (%)			0.905
Endoscopy	17 (27.4)	5 (21.7)	
Abdomen CT	42 (67.7)	17 (73.9)	
Fluoroscopy	3 (4.8)	1 (4.4)	
Time between surgery and leakage diagnosis (days), median (IQR)	9 (7–16)	7 (5–9)	0.062
Leakage size (mm), mean ± SD	8.9 ± 7.3	18.7 ± 9.8	< 0.001
Leakage site, *n* (%)			0.006
Esophagojejunostomy or esophagogastrostomy site	25 (40.3)	18 (78.3)	
Gastroduodenostomy, gastrojejunostomy, or gastrogastrostomy site	14 (22.6)	3 (13.0)	
Duodenal stump or jejunal stump site	23 (37.1)	2 (8.7)	

*CT* computed tomography, *IQR* interquartile range, *SD* standard deviation

AL was most commonly diagnosed using an abdominal CT examination (69.4%). The median time between gastrectomy and AL diagnosis was 8 days (interquartile range [IQR], 6–13 days), and no difference was found regarding this timeframe between the clipping group and stent group (median time, 9 days vs. 7 days; *P* = 0.062). The leakage size was significantly smaller in the clipping group than the stent group (mean leakage size, 8.9 mm vs. 18.7 mm; *P* < 0.001). AL sites were different between the treatment groups (*P* = 0.006). Stent insertions were mostly performed for esophagojejunostomy or esophagogastrostomy AL sites (78.3%), whereas endoscopic clippings with or without detachable snares were performed with similar proportions for esophagojejunostomy or esophagogastrostomy (40.3%) and duodenal or jejunal stump leakage sites (37.1%).

### Outcomes of endoscopic management

The time between AL diagnosis and beginning of endoscopic therapy was longer in the stent group (median 10 days [IQR, 7–22 days]) than in the clipping group (median 5 days [IQR, 0–12 days]; *P* = 0.01). The overall complete closure rate of AL by endoscopic therapy was 80%, and there was no significant difference in the complete closure rate between the clipping group and stent group (79.0% vs. 82.6%; *P* = 0.89; Table 3). The time from the beginning of endoscopic therapy to complete closure of the AL was significantly longer in the stent group than the clipping group (median time, 26 days vs. 13 days, respectively; *P* < 0.001). The groups showed no difference in the total number of endoscopic therapy sessions and the presence of intra-abdominal abscess treated by percutaneous drainage. Because of incomplete leakage closure by the first session of endoscopic therapy, 33 patients (28 in the clipping group and 5 in the stent group) underwent two or more sessions of endoscopic therapies. The clipping group had more patients who needed endoscopic therapy sessions ≥ 2 times than the stent group (45.2% vs. 21.7%; *P* = 0.049).

**Table 3:** Outcomes of endoscopic therapy for anastomotic leakage

Characteristics	Endoscopic therapy		*P* value
	Clips ± snare *N* = 62	Stent *N* = 23	
Time from leakage diagnosis to beginning of endoscopic therapy (days), median (IQR)	5 (0–12)	10 (7–22)	0.01
Combination of endoscopic therapy, *n* (%)			0.023
No	60 (96.8)	19 (82.6)	
Yes	2 (3.2)	4 (17.4)	
Endoscopic therapy session, no (range)	1 (1–11)	1 (1–9)	0.069
Endoscopic therapy session ≥ 2 times, no (%)	28 (45.2)	5 (21.7)	0.049
Intra-abdominal abscess, *n* (%)			0.931
Absent	29 (46.8)	11 (47.8)	
Present	33 (53.2)	12 (52.2)	
Endoscopic therapy result, *n* (%)			0.89
Successful complete closure	49 (79.0)	19 (82.6)	
Partial closure	11 (17.7)	3 (13.0)	
Failed closure	2 (3.2)	1 (4.4)	
Time from beginning of endoscopic therapy and complete closure of leakage (days), median (IQR)	13 (7–24)	26 (18–41)	< 0.001

*IQR* interquartile range

**Fig. 3:**
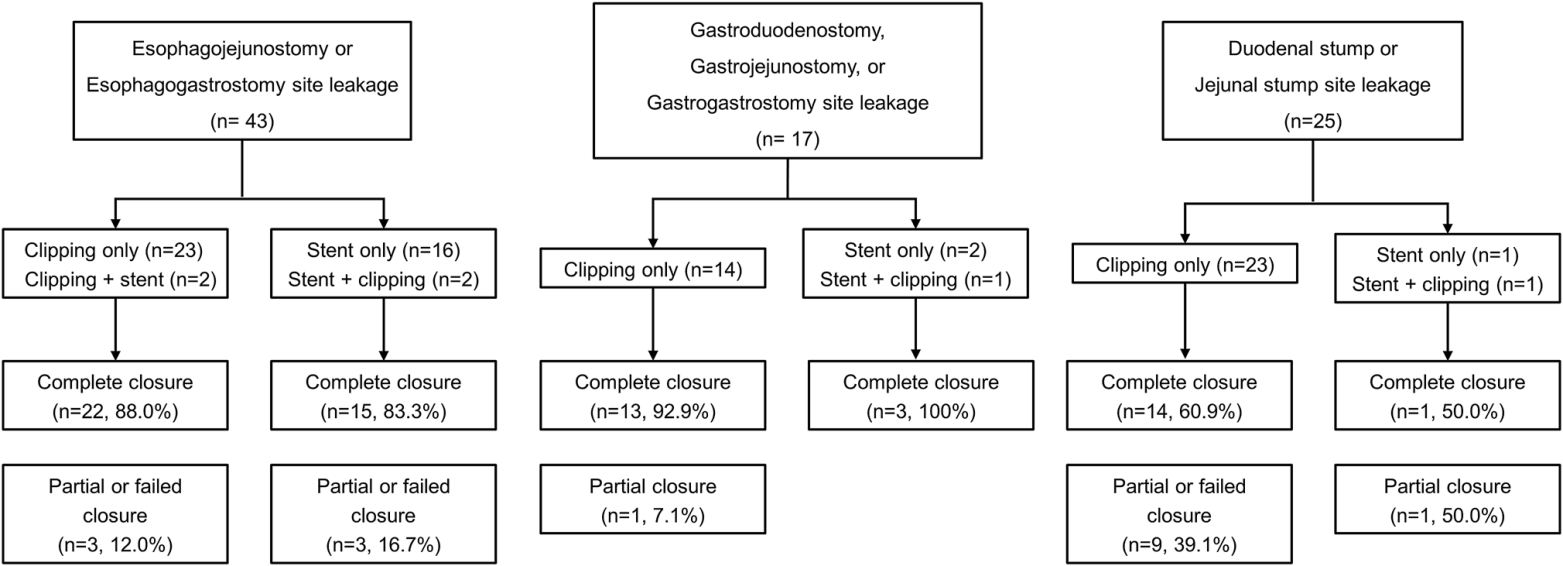
Outcomes of endoscopic therapy according to anastomotic leakage sites after gastrectomy

The complete leakage closure rates were 86.1% (37/43 patients) for esophagojejunostomy or esophagogastrostomy sites, 94.1% (16/17 patients) for gastroduodenostomy, gastrojejunostomy, or gastrogastrostomy sites, and 60.0% (15/25 patients) for duodenal or jejunal stump sites (Fig. 3). In patients with stump site leakages, duodenal stump leakage was detected in 18 patients and jejunal stump in 7 patients. All patients with duodenal stump leakage underwent endoscopic clippings with or without detachable snares, and 12 patients (66.7%, 12/ 18 patients) achieved complete leakage closure. The remaining 6 patients had partial leakage closure, and achieved complete leakage closure after additional conservative treatment. For patients with jejunal stump leakage, stent insertion was performed in 3 patients and endoscopic clippings with or without detachable snares in 4 patients. Only 3 patients (42.9%, 3/7 patients) achieved complete leakage closure. The rate of complete leakage closure was significantly lower for duodenal and jejunal stump sites than the other leakage sites (*P* = 0.026). However, no statistical significance was found in the differences in complete leakage closure rates between the clipping group and stent group at each leakage site. All 14 patients with partial AL closure by endoscopic therapy achieved complete closure after conservative management, including antibiotics and prolonged fasting. Of 3 patients with AL closure failure, 2 patients (2.4%) died 2 months after gastrectomy, and 1 patient achieved complete closure after undergoing a reoperation.

### Factors associated with failure of complete AL closure by endoscopic therapy

Duodenal or jejunal stump leakage (crude odd ratio [cOR], 4.11; *P* = 0.019) and the presence of intra-abdominal abscess (cOR, 5.57; *P* = 0.012) were significantly associated with the failure of complete AL closure in univariate analyses (Table 4). The multivariate analysis showed that independent factors linked to AL closure failure by endoscopic therapy were duodenal or jejunal stump leakage (adjusted OR [aOR], 4.51; *P* = 0.031) and the presence of intra-abdominal abscess (aOR, 4.92; *P* = 0.025). However, the endoscopic modality was not an independent factor associated with unsuccessful AL closures (aOR for stent insertion, 1.38; *P* = 0.672).

**Table 4:** Factors associated with failure of complete closure of anastomotic leakage by endoscopic therapy

Factor	Univariate analysis			Multivariate analysis		
	cOR	95% CI	*P* value	aOR	95% CI	*P* value
Age						
≤ 65 years	1.00					
> 65 years	1.00	0.34–2.94	> 0.99			
Sex						
Male	1.00					
Female	1.09	0.31–3.83	0.896			
Body mass index						
≤ 25 kg/m^2^	1.00					
> 25 kg/m^2^	0.94	0.31–2.85	0.91			
Comorbidity						
Absence	1.00					
Presence	1.00	0.34–2.90	> 0.99			
Tumor stage						
Stage I	1.00					
Stage ≥ II	0.83	0.27–2.51	0.739			
Mode of surgery						
Laparoscopy	1.00					
Open	1.00	0.34–2.90	> 0.99			
Surgery time (minutes)	0.99	0.99–1.00	0.243			
Days between surgery and leakage diagnosis	0.95	0.87–1.03	0.215			
Leakage site						
Esophagojejunostomy or esophagogastrostomy site	1.00			1.00		
Gastroduodenostomy, gastrojejunostomy, gastrogastrostomy, or wedge resection site	0.39	0.04–3.47	0.395	0.53	0.05–5.21	0.587
Duodenal stump or jejunal stump site	4.11	1.27–13.33	0.019	4.51	1.15–17.68	0.031
Leakage size						
≤ 10 mm	1.00					
> 10 mm	1.00	0.33–3.04	> 0.99			
Intra-abdominal abscess						
Absent	1.00			1.00		
Present	5.57	1.47–21.17	0.012	4.92	1.23–19.72	0.025
Days from leakage diagnosis to beginning of endoscopic therapy	1.00	0.97–1.03	0.92			
Modality of endoscopic therapy						
Clips ± snare	1.00					
Stent	0.79	0.23–2.74	0.715	1.38	0.31–6.07	0.672

*aOR* adjusted odd ratio; *cOR* crude odd ratio; *CI* confidence interval

^*^Logistic regression analyses were performed

## Discussion

Previous studies investigating the efficacy of endoscopic management of AL after upper gastrointestinal surgery were limited by small case numbers (fewer than 30) [17–21] and heterogeneous groups of patients who underwent surgery due to differing diagnoses [19, 20]. In this study, we reported the efficacy of endoscopic therapy for a large number of consecutive gastric cancer patients who developed AL after gastrectomy. The rate of complete AL closure by endoscopic therapy was 80%, and endoscopic clippings with or without snares showed comparable success rates of complete leakage closure to that of stent insertion. Duodenal or jejunal stump leakage and the presence of intra-abdominal abscess were associated with incomplete AL closure by endoscopic therapy.

Two recent studies reported therapeutic outcomes comparing two endoscopic therapies for AL after gastrectomy for gastric cancer patients [17, 18]. In a study by Shim et al*.* [18], stent insertion using SEMS showed a significantly higher success rate of AL sealing after one attempt than endoscopic clippings with or without a detachable snare (80.0% vs. 28.6%, respectively). There was no difference in the success rate regarding multiple endoscopic sessions between the two endoscopic therapies (80.0% for stent insertion vs. 64.3% for clippings; *P* = 0.653). However, this study was limited by its small case size (only 27 patients who had esophagojejunostomy AL after gastrectomy were included). Another study reported a relatively high success rate of endoscopic clippings to treat AL after total or subtotal gastrectomy in 20 gastric cancer patients [19]. This study was also limited by its small case size.

Reported success rates of endoscopic therapy for AL in patients who underwent gastrectomy are between 59 and 95% [17–19]. However, these studies did not compare the outcomes of different AL sites. In the present study, the total success rate of complete AL closure was 80%, which is similar to previous studies. Success rates also varied among different AL sites, with duodenal or jejunal stump leakage showing a lower complete closure rate (60%) than ALs of the esophagus (86%) and stomach (94%). Moreover, stump leakage was an independent factor associated with the failure of endoscopic therapy for AL. The lower success rate of endoscopic therapy for stump leakages may be due to the difficulty of approaching this region using endoscopy as a result of access issues such as limited space and stump opening angulation.

Moreover, the characteristics of the AL site affected the type of endoscopic therapy selected in this study. Endoscopic clippings with or without detachable snares were used more frequently for AL at stomach and stump sites than stents. Regarding AL sites of the stomach, as in the case of gastrojejunostomy or gastrogastrostomy, stent insertion appeared to be ineffective for two main reasons: the anastomotic lumen was so large that the stent could not fully cover the leakage site, or the stent readily migrated from the placed site.

In addition, stent insertion was generally not performed for stump leakages because these locations did not have suitable anatomy for stent placement. However, stent insertion was used more frequently for cases of large esophageal AL because of easier technical access and a smaller number of endoscopic therapy sessions than clippings, as shown in a previous study [18]. These findings suggest that delivering tailored endoscopic therapy according to the leakage site characteristics may be needed to achieve better outcomes.

Surgical correction with primary closure was considered for duodenal stump leakages, but reoperation was not recommended due to its high mortality risk of 20 to 28% [11–13]. Thus, the least invasive method of treatment was performed to manage duodenal stump leakage, including conservative treatment [11, 12], percutaneous drainage [12, 25], or endoscopic therapy [26, 27]. Despite encouraging preliminary evidence describing the efficacy of the endoscopic treatment of AL, the outcomes have not been investigated adequately, and only a case report has been published. In the present study, stump leakage sites were implicated as an independent risk factor for the failure of complete leakage closure by endoscopic therapy. The success rate of complete closure in this study was relatively high (60% of 25 patients) despite technical and anatomical difficulties hindering the endoscopic approach for stump sites.

Intra-abdominal abscess can occur as a result of AL after gastrectomy and manifests as abdominal pain, fever, and leukocytosis. Furthermore, this complication may cause a delay of complete AL closure. Thus, intravenous antibiotics and external drainage are needed to treat intra-abdominal abscess after gastrectomy. Intra-abdominal abscess occurred in 53% of AL after gastrectomy in this study and was another significant risk factor for the failure of complete leakage closure by endoscopic therapy. This result suggests that earlier and more intensive interventions should be performed to achieve complete AL closure by endoscopic therapy, especially in patients with intra-abdominal abscess.

**Fig. 4:**
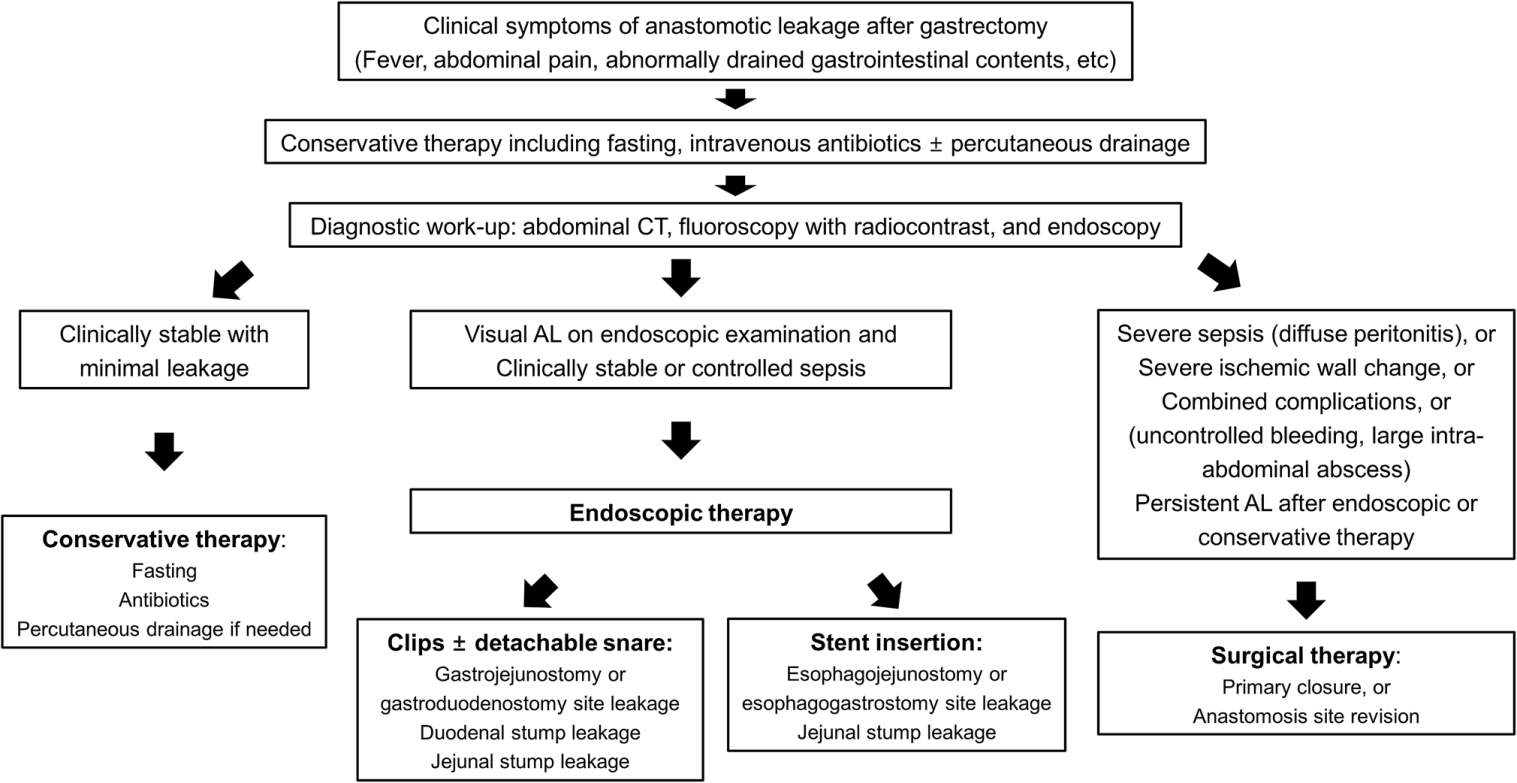
Suggested treatment algorithm for the management of AL developing after gastrectomy

Based on our study findings, we suggest a treatment algorithm for the management of AL developing after gastrectomy (Fig. 4). After the diagnostic work-up with abdominal CT scan, fluoroscopy with radiocontrast and endoscopy, the therapy provided is determined according to patient clinical status. Conservative therapy is provided to patients who are clinically stable and had a minimal AL not visible on endoscopic examination. Endoscopic therapy is performed in patients with a visible AL who had controlled sepsis or whose clinical status is stable. Endoscopic therapy modalities are selected according to the AL site. Meanwhile, surgical therapy is performed in patients with following conditions: severe septic condition, severe ischemic wall change, other combined complications requiring surgical treatment including uncontrolled bleeding or large intra-abdominal abscess, or persistent AL after conservative or endoscopic therapy.

The strengths of this study include its large cohort size and outcome analysis based on the stratification of different AL sites, especially for stump leakage sites. However, it has several limitations. First, this study was retrospectively conducted with patient data from a single institution. Second, the choice of endoscopic technique (clippings vs. stents) was at the discretion of the endoscopist. In addition, therapeutic options of AL (conservative vs. endoscopic vs. surgical) were determined according to patients’ clinical status and extent of AL. These may have resulted in selection bias.

In conclusion, the results of this study strongly indicate that endoscopic therapy is effective for the treatment of AL post-gastrectomy in gastric cancer patients. Therefore, it may be considered as a primary treatment option in treating ALs after gastrectomy in gastric cancer patients.

## Abbreviations

AL: Anastomotic leakage
ASA: American Society of Anesthesiologists
IRB: Institutional Review Board
CT: Computed tomography
SEMS: Self-expandable metal stent
